# Supplementary material for: Insight into the Amelioration Effect of Nitric Acid-Modified Biochar on Saline Soil Physicochemical Properties and Plant Growth
Source: Plants (Basel). 2024 Dec 6;13(23):3434. doi: 10.3390/plants13233434 (PMC11644791; doi:10.3390/plants13233434)
Supplement: Supplementary file 1 [file plants-13-03434-s001.zip › plants-3339226-supplementary.pdf]

Table S1 Brunauer Emmet Teller (BET) and Barrett Joyner Helenda (BJH) calculate the specific surface area, pore volume, and pore size of BC and HBC samples.

	Specific surface area (m^2/g)	Pore volume (cm^3/g)	Average pore diameter (cm^3/g)
BC	25.6082	0.010231	0.003533
HBC	66.3699	0.02756	0.015361

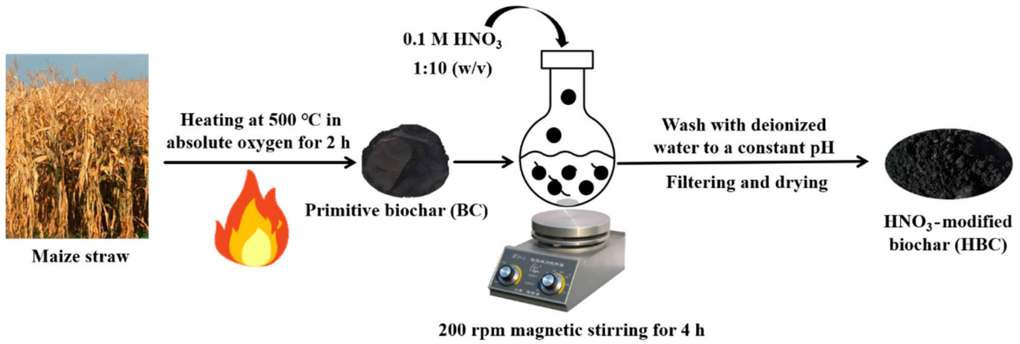

Figure S1 Preparation process of nitric acid modified biochar. 10 g of BC to 100 mL of 0.1 M HNO_3 solution, and stir the suspension at 200 rpm for 4 h at 25 ℃. Filter and separate the acidified biochar from the solution, and then thoroughly wash it with deionized water until the pH remains constant. Finally, the obtained modified biochar was dried in a blast oven at 60 °C to obtain HBC.

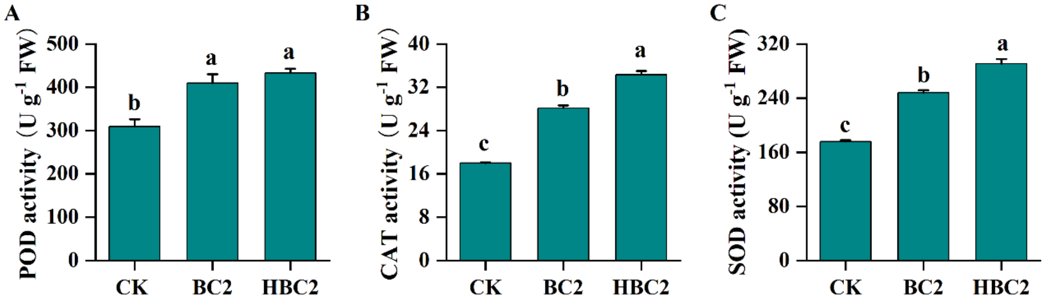

Figure S2 Effects of BC and HBC on the antioxidant enzyme activity of the leaf of pakchoi seedlings. Fig-A: POD activity; Fig-B: CAT activity; Fig-C: SOD activity. Different letters (a, b, c) represent significant differences at 95 % probability level.
